# B cell-activating factor of the tumor necrosis factor family (BAFF) is expressed under stimulation by interferon in salivary gland epithelial cells in primary Sjögren's syndrome

**DOI:** 10.1186/ar1912

**Published:** 2006-02-28

**Authors:** Marc Ittah, Corinne Miceli-Richard, Jacques- Eric Gottenberg, Frédéric Lavie, Thierry Lazure, Nathalie Ba, Jérémie Sellam, Christine Lepajolec, Xavier Mariette

**Affiliations:** 1 Rhumatologie, Institut Pour la Santé et la Recherche Médicale (INSERM) U 802, Hôpital Bicêtre, Assistance Publique-Hôpitaux de Paris (AP-HP), Université Paris-Sud 11, 78 rue du Général Leclerc, 94275 Le Kremlin Bicêtre, France; 2 Anatomopathologie, Hôpital de Bicêtre, AP-HP, 78 rue du Général Leclerc, 94275 Le Kremlin Bicêtre, France; 3 Oto-rhino-laryngologie, Hôpital de Bicêtre, AP-HP, 78 rue du Général Leclerc, 94275 Le Kremlin Bicêtre, France

## Abstract

B cell-activating factor (BAFF) has a key role in promoting B-lymphocyte activation and survival in primary Sjögren's syndrome (pSS). The cellular origin of BAFF overexpression in salivary glands of patients with pSS is not fully known. We investigated whether salivary gland epithelial cells (SGECs), the main targets of autoimmunity in pSS, could produce and express BAFF. We used quantitative RT-PCR, ELISA and immunocytochemistry in cultured SGECs from eight patients with pSS and eight controls on treatment with IL-10, tumor necrosis factor α (TNF-α), IFN-α and IFN-γ. At baseline, BAFF expression in SGECs was low in pSS patients and in controls. Treatment with IFN-α, IFN-γ and TNF-α + IFN-γ increased the level of BAFF mRNA in pSS patients (the mean increases were 27-fold, 25-fold and 62-fold, respectively) and in controls (mean increases 19.1-fold, 26.7-fold and 17.7-fold, respectively), with no significant difference between patients and controls. However, in comparison with that at baseline, stimulation with IFN-α significantly increased the level of BAFF mRNA in SGECs of pSS patients (*p *= 0.03) but not in controls (*p *= 0.2), which suggests that SGECs of patients with pSS are particularly susceptible to expressing BAFF under IFN-α stimulation. Secretion of BAFF protein, undetectable at baseline, was significantly increased after IFN-α and IFN-γ stimulation both in pSS patients (40.8 ± 12.5 (± SEM) and 47.4 ± 18.7 pg/ml, respectively) and controls (24.9 ± 8.0 and 9.0 ± 3.9 pg/ml, respectively), with no significant difference between pSS and controls. Immunocytochemistry confirmed the induction of cytoplasmic BAFF expression after stimulation with IFN-α and IFN-γ. This study confirms the importance of resident cells of target organs in inducing or perpetuating autoimmunity. Demonstrating the capacity of SGECs to express and secrete BAFF after IFN stimulation adds further information to the pivotal role of these epithelial cells in the pathogenesis of pSS, possibly after stimulation by innate immunity. Our results suggest that an anti-BAFF therapeutic approach could be particularly interesting in pSS.

## Introduction

Primary Sjögren's syndrome (pSS) is a prototypical autoimmune disorder characterized by lymphocytic infiltration of salivary and lachrymal glands leading to xerostomia and keratoconjunctivitis sicca. Polyclonal B cell activation and systemic production of autoantibodies are the main laboratory findings characterizing pSS [1]. Patients with pSS are at increased risk for the development of B cell non-Hodgkin's lymphoma, and some evidence exists that such lymphomas [2,3] arise from autoreactive B cells [4-6].

Recruitment of activated and memory B cells in salivary gland infiltrates [7], germinal center formation in 20 to 25% of patients, and local secretion of autoantibodies [8] demonstrate the pathogenic role *in situ *of B cell activation in pSS. Increased expression of a newly described cytokine, termed B cell-activating factor (BAFF) or B-lymphocyte stimulator (BLyS) [9-12], might explain this pathogenic B cell activation in several systemic autoimmune diseases including pSS.

BAFF has a crucial role in B cell maturation [13-15], plasma cell survival [15], antibody response promotion [16] and immunoglobulin-class switch recombination [17]. Interestingly, for reasons that are not fully understood, autoreactive B cells depend on BAFF for survival more than alloreactive B cells do [18,19]. The involvement of BAFF in the pathogenesis of autoimmune diseases is well illustrated by BAFF overexpression in mice models, which leads to autoimmune disease mimicking rheumatoid arthritis (RA), systemic lupus erythematosus (SLE) and pSS, as well as a twofold increase in occurrence of B cell lymphoma [13]. In humans, an increased serum level of BAFF was reported in patients with RA [20,21] and SLE [22,23], but the more consistent findings concerned pSS, with an increase in BAFF level reported in all four published surveys of patients with pSS [24-27]. Moreover, we demonstrated in pSS a correlation between the serum level of BAFF and serum level of immunoglobulins and titers of autoantibodies [25,28].

Using immunohistochemistry, we and others have shown increased expression of BAFF in salivary glands of patients with pSS [24,29,30]. We recently extended these results by demonstrating a threefold increase in BAFF mRNA level in the two main target organs of pSS salivary glands and the ocular surface [31]. However, the cellular origin of BAFF expression in salivary glands of patients with pSS is not well understood. Indeed, monocytes and myeloid dendritic cells, the main cell types involved in the physiological expression of BAFF [32], are not present in large amounts in salivary glands of patients with pSS. Using immunohistochemistry, we localized BAFF expression in the T cell infiltrate and ductal epithelial cells [29]. However, we could not eliminate the possibility that this finding was due to the passive fixation of BAFF on its receptor.

Glandular epithelial cells are the main target cells of autoimmunity in pSS [33], currently considered to be an autoimmune epithelitis [34]. These cells, after exogenous aggression, possibly of viral origin [35], express co-stimulation molecules [36-38] and lymphoid chemokines [39] and are suitably equipped to present autoantigens, which suggests that salivary gland epithelial cells (SGECs) can act as non-professional antigen-presenting cells [40]. Thus, we proposed that SGECs could also express BAFF in pSS. To avoid the limitation of immunohistochemical studies, potentially showing passive BAFF fixation on one of its receptors rather than the cellular production of BAFF, we investigated BAFF mRNA expression in salivary gland cell lines. We then investigated BAFF mRNA expression in SGECs from patients with pSS and controls, and BAFF protein secretion in supernatants from these cell cultures. We evaluated the contribution of different patterns of cytokine environment on BAFF mRNA and protein expression, using stimulations with various cytokines known to have a pathogenic role in pSS. Our results demonstrate the inducible expression of BAFF mRNA and BAFF protein under stimulation by IFN in SGECs, which might have a key pathogenic role in pSS autoimmune epithelitis.

## Materials and methods

### Patients

Eight female patients with pSS (mean age 43 years; range 35 to 59 years) referred to the Department of Rheumatology, Bicêtre Hospital, France, were enrolled in the study. pSS was defined in accordance with the American/European consensus group (AECG) criteria [41]. Seven of eight patients had serum anti-SSA antibodies, and four also had anti-SSB antibodies. All except one had a positive lip biopsy (Chisholm score of 3 or 4). The only patient with a negative lip biopsy also had anti-SSA antibodies and fulfilled AECG criteria. No patients had evidence of other connective tissue disease. Biopsy specimens of minor salivary glands were obtained from eight control subjects (seven women and one man, mean age 54 years; range 35 to 79 years) with sicca symptoms without autoantibodies or lymphoid infiltrates on lip biopsy. The study received approval from the local ethics committee, and informed consent was obtained from all study subjects.

### Reagents

DMEM, Ham's F-12 and DMEM/F-12 were from Invitrogen (Cergy Pontoise, France). FCS and 0.125% trypsin-EDTA were from Seromed (Berlin, Germany). Hydrocortisone was from Pharmacia (Guyancourt, France). Insulin was from Novo Nordisk A/S (Denmark). Epidermal growth factor was from BD Bioscience (Le Pont de Claix, France). Recombinant human IFN-γ (IFN-γ), IFN-α and IL-10 were purchased from R&D Systems (Lilles, France). Recombinant human tumor necrosis factor-α (TNF-α) and cytokeratin 19 were from Sigma-Aldrich (Saint Quentin Fallavier, France). Cytokeratin 7 was from Biogenex (Antony, France). Cytokeratins 20 and 903, and CD45 and EnVision Detection Kit peroxidase/diaminobenzidine, rabbit/mouse were from DakoCytomation (Trappes, France). Myeloperoxidase (MPO) was from Novocastra (Newcastle, UK). Rat anti-human BAFF (Buffy-2) was kindly provided by Pascal Schneider (Apotech).

### Cell lines

HSG is a cell line derived from neoplastic epithelial duct cells of the human salivary gland (a gift of Bruce Baum and Marc Kok (U.S. National Institutes of Health)), grown in DMEM/F-12 supplemented with 10% FCS, penicillin (100 IU/ml) and streptomycin (100 μg/ml). Human erythroleukemia K562 cells stably expressing BAFF were grown in RPMI medium supplemented with 10% FCS, penicillin (100 IU/ml) and streptomycin (100 μg/ml). All cell lines were incubated at 37°C under 5% CO_2_.

### Cultures of SGECs and treatment

Primary cultures of SGECs were established from minor salivary glands as described [42]. In brief, each lobule was cut into small fragments and set in six 75 cm^2 ^flasks with basal epithelial medium (a 3:1 mixture of Ham's F-12 and DMEM) supplemented with 2.5% FCS, epidermal growth factor (10 ng/ml), hydrocortisone (0.4 μg/ml), insulin (0.5 μg/ml), penicillin (100 IU/ml) and streptomycin (100 μg/ml) and incubated at 37°C under 5% CO_2_. After 4 to 5 weeks of culture, at 70 to 80% confluence, cells were stimulated with IL-10 (100 ng/ml), IFN-α (2,400 U/ml), IFN-γ (5 ng/ml), TNF-α (1 ng/ml) or IFN-γ (5 ng/ml) + TNF-α (1 ng/ml) for 2 days for real-time quantitative RT-PCR and ELISA. Cells were then dissociated with 0.125% trypsin-EDTA solution.

### Real-time quantitative RT-PCR

Total RNA was isolated from epithelial cells with use of the RNeasy Mini kit from Qiagen (Courtaboeuf, France). cDNA synthesis involved the use of Enhanced Avian HS RT-PCR Kit from Sigma-Aldrich (Saint Quentin Fallavier, France). BAFF and β-actin cDNA levels were determined by use of Light Cycler-based kinetic quantitative PCR (Roche Diagnostics, Meylan, France). BAFF and β-actin PCR products were detected by the use of LightCycler FastStart DNA Master SYBR Green I (Roche Diagnostics). To correct for variations in mRNA recovery and reverse transcription yield, the amount of BAFF cDNA was normalized with β-actin. Results were expressed as an increase in normalized values over that observed with untreated cells. Amplification primers for the human genes were as follows: BAFF, 5'-TGAAACACCAACTATACAAAAAG-3' and 5'-TCAATTCATCCCCAAAGACAT-3'; β-actin, 5'-GCTGTGCTACGTCGCCCT-3' and 5'-AAGGTAGTTTCGTGGATGCC-3'. Primers were designed to be specific to full-length BAFF, excluding any amplification of delta-BAFF, an alternative splice variant lacking exon 3. Quantitative PCR runs were considered only if amplification efficiencies were high (slopes ranging from -3.2 to -3.8). Each sample was processed in duplicate, with initial incubation at 96°C for 10 minutes, and thermal conditions followed 40 cycles of 95°C for 10 seconds, 60°C for 15 seconds, and 72°C for 20 seconds. For each run, serially diluted cDNA from K562 cells was used for quantitative standards. We determined the cell equivalence number of BAFF and β-actin mRNA in each sample in accordance with the standard curve generated from values obtained with K562. The unit number showing relative BAFF mRNA level in each sample was determined as a value of BAFF cell equivalence normalized with β-actin cell equivalence. Melting-curve analysis was performed to assess the specificity of PCR product.

### Immunocytochemistry

SGECs were pelleted and fixed in AFA (alcohol, acetic acid and formaldehyde) solution and embedded in paraffin wax. Cell sections were dewaxed in 100% xylene and rehydrated by serial incubations in ethanol, then water and PBS. Cell sections were pretreated by microwave heating in citrate buffer, pH 7.3, for 15 minutes. After incubation for 10 minutes at room temperature with bovine serum albumin in PBS, slides were incubated for 30 minutes in a humid chamber at 4°C with 20 μg/ml mouse anti-human cytokeratin 7, cytokeratin 19, cytokeratin 20, cytokeratin 903, CD45, CD20, CD3, smooth muscle actin and MPO. For BAFF stainings, slides were incubated overnight with 20 μg/ml rat anti-human BAFF (Buffy-2). Specimens were treated with 3% H_2_O_2 _in PBS for 5 minutes to inactivate endogenous peroxidase activity. Then slides were incubated at room temperature for 30 minutes each, with the use of an EnVision Detection Kit peroxidase/diaminobenzidine, rabbit/mouse. Staining involved the use of the 3-amino-9-ethylcarbazole (AEC) chromogen (DakoCytomation). The positive control for BAFF staining was a tonsil section. The negative control consisted of staining SGECs with rat immunoglobulins by using the EnVision Detection Kit.

### Detection and quantification of BAFF secretion

BAFF levels in the supernatants of primary cultures of unstimulated or stimulated SGECs were determined by using an ELISA kit from R&D Systems.

### Data analysis and statistics

Results are shown as means ± SEM. Statistical comparison involved the Mann-Whitney *U *test and the Wilcoxon matched-pairs test with use of Analyse-it software (Analyse-it Software Ltd, Leeds, UK) for Microsoft Excel.

## Results

### BAFF expression in salivary gland cell lines

**Figure 1:**
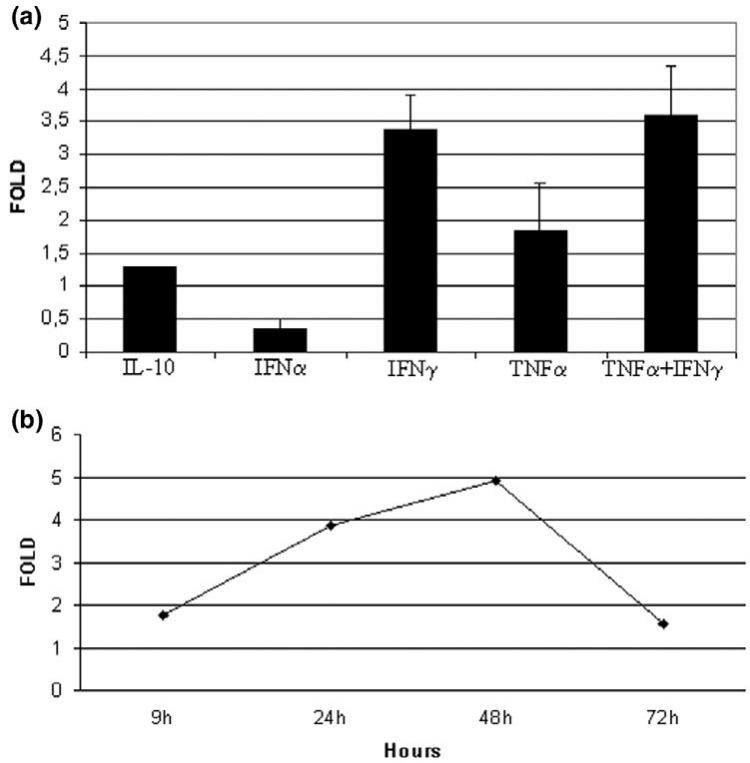
BAFF mRNA in the HSG cell line after stimulation by cytokines. **(a) **RT-PCR results showing expression of B cell-activating factor (BAFF) mRNA by the HSG cell line after 48 hours of stimulation with IL-10 (100 ng/ml), IFN-α (2,400 U/ml), IFN-γ (5 ng/ml), tumor necrosis factor (TNF)-α (1 ng/ml) and IFN-γ (5 ng/ml) + TNF-α (1 ng/ml). **(b) **Time course of induction of BAFF mRNA in the HSG cell line 9, 24, 48 and 72 hours after stimulation with IFN-γ (5 ng/ml). Error bars indicate SEM.

Quantitative RT-PCR detected low BAFF gene expression at baseline in the HSG cell line. A significant increase was observed after stimulation with IFN-γ and IFN-γ + TNF-α for 48 hours (the mean increases were 3.4-fold and 3.6-fold, respectively). No significant change was observed with IL-10, IFN-α or TNF-α (Figure 1a). Next we investigated the time course of BAFF mRNA induction. The level of BAFF mRNA peaked 48 hours after the addition of IFN-γ (Figure 1b). Subsequently, these positive results in epithelial cell lines led us to determine whether they could be extended to primary epithelial cells in patients with pSS and controls.

### Epithelial origin of salivary gland cells

**Figure 2:**
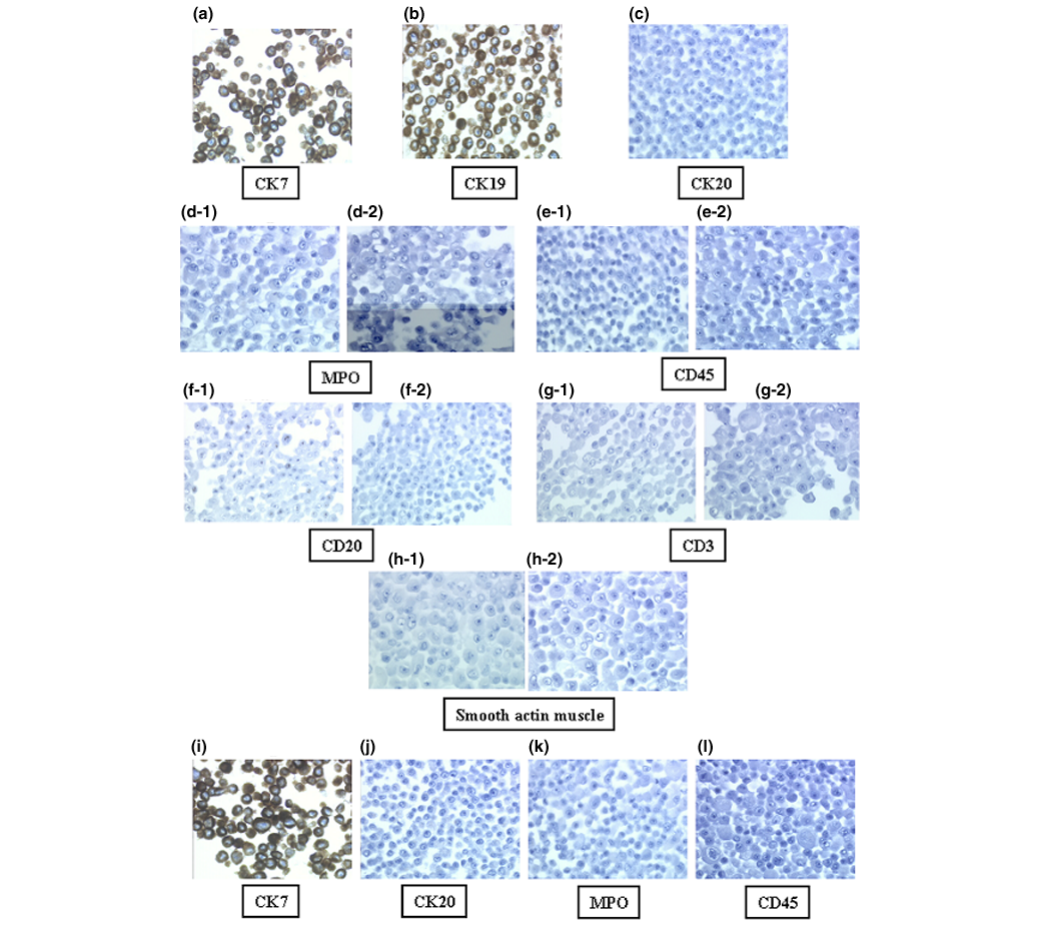
Immunocytochemical analysis of salivary epithelial cells. Cells were obtained from minor salivary glands **(a–e) **and the HSG cell line **(f–i)**. Positive staining for cytokeratin 7 (a) and cytokeratin 19 (b) and the absence of staining with cytokeratin 20 (c) indicates the specificity of ductal epithelial cell origin. The absence of staining for myeloperoxidase (MPO) (d), CD45 (e), CD20 (f), CD3 (g) and smooth muscle actin (h) before (subpanels 1 in (d–h)) and after (subpanels 2 in (d–h)) stimulation with IFN-α excludes the possibility of contamination with myeloid cell. Positive staining with cytokeratin 7 **(i) **and the absence of staining with cytokeratin 20 **(j) **indicate the ductal epithelial origin of HSG cell lines. Negative staining with myeloperoxydase (MPO) **(k) **and CD45 **(l) **excludes the possibility of contamination with myeloid cells.

To exclude cell dedifferentiation during primary culture and after cytokine stimulation, morphological characteristics and cytokeratin 7 staining for ductal SGECs [43] were evaluated in cells from patients with pSS and from controls. All tested samples were 95 to 100% positive for cytokeratin 7, cytokeratin 19 and cytokeratin 903 but not for cytokeratin 20, which confirmed the ductal epithelial origin of these cells (Figure 2a–c). Moreover, complementary staining with MPO, CD20, CD3, CD45 and smooth muscle actin excluded the possibility of contamination with myeloid cells, B cells, T cells or myoepithelial cells (Figure 2d–h). The HSG cell line had exactly the same staining pattern as epithelial cells from patients (positive for cytokeratin 7 and negative for cytokeratin 20, MPO and CD45; Figure 2i–l).

### Expression of BAFF mRNA in ductal SGECs

**Figure 3:**
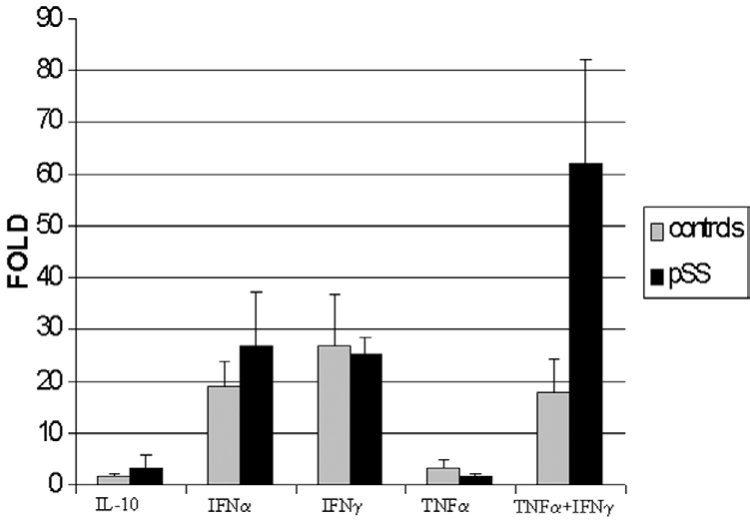
Induction of BAFF mRNA by epithelial cells from minor salivary glands. Results are from seven patients with pSS and from seven controls, 48 hours after stimulation with IL-10 (100 ng/ml), IFN-α (2,400 U/ml), IFN-γ (5 ng/ml), tumor necrosis factor (TNF)-α (1 ng/ml) and IFN-γ (5 ng/ml) + TNF-α (1 ng/ml). All samples were processed in duplicate. Error bars indicate SEM. BAFF, B cell-activating factor.

At baseline, BAFF expression in SGECs was low in patients with pSS and not significantly different from that of controls (Figure 3 and Table 1). Treatment with IFN-α, IFN-γ and TNF-α + IFN-γ increased the level of BAFF mRNA in patients with pSS (mean increases 27-fold, 25-fold and 62-fold, respectively) and in controls (mean increases 19.1-fold, 26.7-fold and 17.7-fold, respectively), with no significant difference between patients and controls (*p *= 0.5, 0.8 and 0.07, respectively). However, compared with that at baseline, the level of BAFF mRNA was significantly increased with IFN-α in SGECs of pSS patients (*p *= 0.03) but not in those of controls (*p *= 0.2), which suggests that SGECs of patients with pSS are particularly susceptible to expressing BAFF under stimulation with IFN-α. IFN-γ significantly induced BAFF expression in patients with pSS and in controls (*p *= 0.008 and 0.03, respectively). The effect on BAFF expression of stimulation of pSS-patient cells with IFN-γ + TNF-α was not significantly different from that of stimulation with IFN-γ alone (*p *= 0.15; Figure 3). No change in BAFF expression was observed in cells stimulated by IL-10 or TNF-α.

### Expression of BAFF protein in ductal SGECs

**Figure 4:**
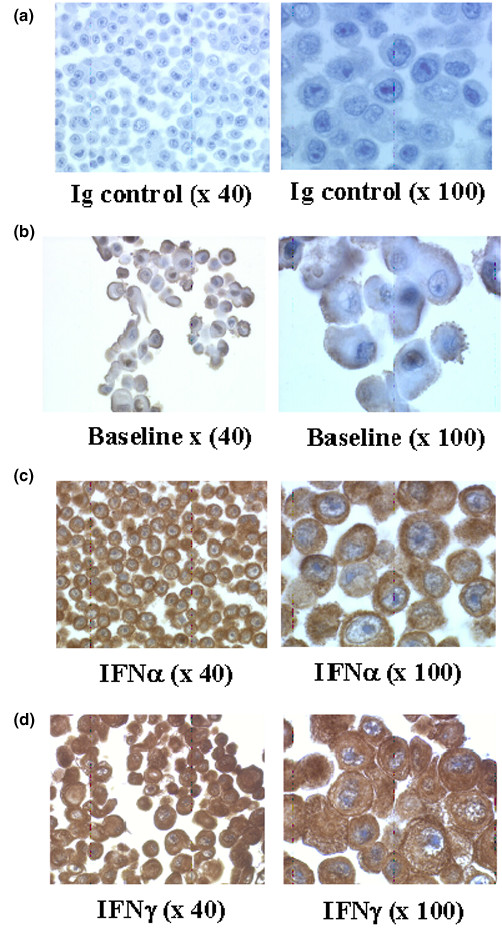
Immunocytochemical analysis of BAFF expression in salivary epithelilal cells from minor salivary glands. Positive staining for B cell-activating factor (BAFF) 48 hours after stimulation with IFN-α (2,400 U/ml) **(c) **and with IFN-γ (5 ng/ml) **(d) **was markedly enhanced compared with baseline **(b)**. **(a) **Negative control with polyclonal rat immunoglobulin.

Immunocytochemistry analysis in pSS-patient SGEC cell cultures showed a slight positive staining at baseline (Figure 4b) that was markedly enhanced after 48 hours with IFN-α (Figure 4c) and IFN-γ (Figure 4d).

### BAFF production by supernatant of primary epithelial cell culture

**Figure 5:**
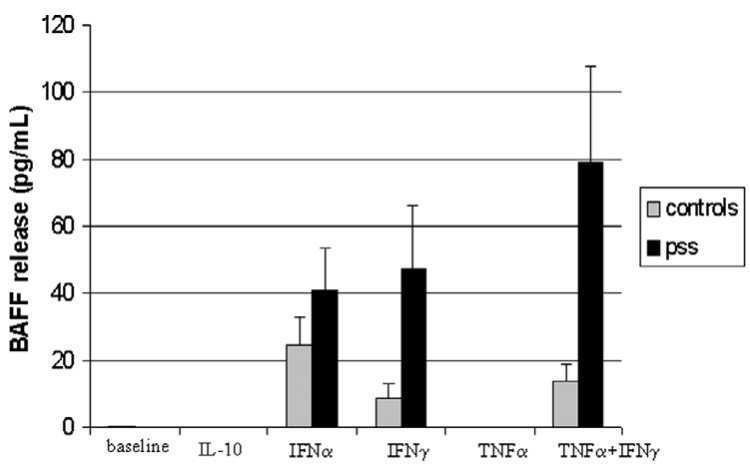
ELISA results of BAFF secretion by salivary epithelial cells from minor salivary glands. Results are from eight patients with pSS and from eight controls. Supernatant of culture was harvested 48 hours after stimulation with IL-10 (100 ng/ml), IFN-α (2,400 U/ml), IFN-γ (5 ng/ml), tumor necrosis factor (TNF)-α (1 ng/ml) and IFN-γ (5 ng/ml) + TNF-α (1 ng/ml). Error bars indicate SEM. All samples were processed in duplicate. BAFF, B cell-activating factor.

Because BAFF can be produced as a soluble protein, we investigated whether soluble forms of BAFF could be secreted by epithelial cells. At baseline and after stimulation with IL-10 and with TNF-α, ELISA detected no soluble BAFF in cells from patients with pSS and from controls (Figure 5). Soluble BAFF was detected by ELISA after stimulation with IFN-α in all tested samples and after stimulation with IFN-γ in all samples except three controls. Secretion of BAFF protein was significantly increased after stimulation with IFN-α and IFN-γ both in patients with pSS (40.8 ± 12.5 pg/ml, *p *= 0.03, and 47.4 ± 18.7 pg/ml, *p *= 0.02, respectively) and in controls (24.9 ± 8.0 pg/ml, *p *= 0.04, and 9.0 ± 3.9 pg/ml, *p *= 0.04, respectively). After stimulation with IFN-α and IFN-γ, pSS patients and controls showed no difference in BAFF protein secretion (*p *= 0.90 and *p *= 0.22, respectively). After stimulation with IFN-γ + TNF-α, BAFF secretion showed a trend to a greater increase in patients with pSS than in controls (*p *= 0.09; Figure 5). Patients with PSS showed a trend toward potentiation of the effect of IFN-γ when combined with TNF-α: the BAFF level with IFN-γ + TNF-α was 79.3 ± 28.3 pg/ml, compared with 47.4 ± 18.7 pg/ml with IFN-γ alone (*p *= 0.08; Figure 5).

## Discussion

Analysis of target organs of autoimmunity might help provide insights into the pathogenesis of these diseases. We demonstrate for the first time that BAFF, a critical molecule involved in B cell survival, can be induced in SGECs under stimulation with IFN at the mRNA and protein levels. Contamination by myeloid cells, which could have been responsible for BAFF secretion, was eliminated both in epithelial-cell cultures from patients and in the HSG cell line, as seen by the total absence of staining with myeloid markers in immunocytochemistry and the 100% positivity with epithelial markers. Patterns of BAFF mRNA expression as assessed by quantitative RT-PCR, and BAFF secretion as assessed by ELISA after stimulation with IFN-α, IFN-γ, and IFN-γ + TNF-α, were similar. Immunocytochemistry confirmed the induction of BAFF after stimulation with IFN-α or IFN-γ. Thus, we demonstrated a significant increase in secreted BAFF protein. The functional effect of BAFF on B cell survival was demonstrated previously [44].

The present results confirm the recent demonstration in RA [45] and multiple sclerosis [46] that resident cells of target organs of autoimmunity could be induced to express BAFF under environmental conditions. Our results also emphasize that BAFF induction by cytokines is cell and disease dependent. IL-10, a cytokine involved in BAFF stimulation in monocytes and myeloid dendritic cells [47], did not influence BAFF expression in SGECs, and neither did TNF-α, which was recently reported to induce BAFF expression in fibroblast-like synoviocytes of patients with RA [45]. Our results agree with findings of the absence of a major role of TNF-α in the pathogenesis of pSS, as illustrated by the lack of efficacy of TNF-α blockers in this disease [48]. However, we observed a non-significant trend suggesting a synergistic effect of IFN-γ and TNF-α on the expression of both BAFF mRNA and protein. A similar result was reported in synoviocytes from patients with RA [45] and in astrocytes from patients with multiple sclerosis [46]. This possible synergistic effect could be due to an induction of IFN-γ receptors by stimulation with TNF-α [49].

Interestingly, IFN-α and IFN-γ increased BAFF mRNA and protein levels in epithelial cells both from patients with pSS and from controls but to a higher level in cells from patients. Indeed, the increased BAFF mRNA level from baseline on stimulation with both IFNs and IFN-γ + TNF-α reached statistical significance in patients, whereas only IFN-γ stimulation significantly increased the BAFF mRNA level in controls (Figure 3 and Table 1). Stimulation with both IFNs and with IFN-γ + TNF-α significantly increased BAFF protein level in the supernatants of epithelial cells in pSS patients and controls, as revealed by ELISA (Figure 5).

The possibly higher sensitivity to IFN stimulation, as revealed by quantitative PCR, might be an intrinsic property of epithelial cells in pSS and could be related to genetic factors. The action of IFN-α on BAFF expression was previously studied only on follicular dendritic cells of the germinal center [17]. IFN-α has a pivotal pathogenic role in SLE [50] and could also be involved in the pathogenesis of pSS. Recently, a study described IFN-α-positive cells in salivary gland infiltrates of patients with pSS [51]. In addition, an IFN signature was demonstrated in two microarray studies of salivary glands of patients with pSS [31,52]. Moreover, Bave and colleagues showed that the combination of anti-SSA antibodies and apoptotic cells have a key role in the induction of IFN-α by peripheral blood mononuclear cells in pSS [51]. Thus, in the salivary glands of patients with pSS, the combination of apoptotic bodies from epithelial cells and anti-SSA antibodies could induce IFN-α production by infiltrating cells, which could then induce BAFF expression by epithelial cells. Of course, another source of IFN-α could be local viral infection, which has frequently been suspected to induce pSS but never with a definitive demonstration [53-56]. Whatever the cause of a possible increase in IFN in the target organs of pSS, our results suggest that SGECs of patients with pSS are particularly susceptible to BAFF induction by IFN. Interestingly, BAFF can be also increased in the ocular surface, another epithelial target of autoimmunity in pSS: indeed, we found in conjunctival smears a threefold increase in BAFF mRNA level in patients with pSS in comparison with controls [31].

Our results add to the understanding of the pathogenic involvement of SGECs in pSS. Such cells can not only express and present autoantigens but can also concomitantly activate B cells by the local secretion of BAFF. BAFF overexpression might have a pathogenic role in lymphomas, because increased BAFF expression was observed in some lymphomas, and an increased BAFF level in serum was associated with a worse prognosis in patients with lymphomas [57]. Thus, local secretion of BAFF by epithelial cells might also explain why lymphomas originate from the clonal transformation of salivary gland autoreactive B cells in patients with pSS [4,5].

## Conclusion

We have shown a new capacity of SGECs to express and secrete BAFF after stimulation by IFN. This peculiar property of epithelial cells is enhanced in patients with pSS and confirms the importance of resident cells of target organs in inducing or perpetuating autoimmunity and the pivotal role of epithelial cells in the pathophysiological aspects of pSS. To a larger extent, our results suggest a complex modulation of BAFF expression depending on the pattern of cytokines involved in each autoimmune disease and on the cytokine sensitivity of resident cell types present in target organs. In pSS, BAFF could be a mediator between innate and adaptative immunity, leading to the stimulation of autoreactive B cells. Last, as suggested [58], our results give some arguments in favor of a therapeutic effect of an anti-BAFF approach in pSS.

## Abbreviations

BAFF = B cell-activating factor; DMEM = Dulbecco's modified Eagle's medium; ELISA = enzyme-linked immunosorbent assay; FCS = fetal calf serum; IFN = interferon; IL = interleukin; MPO = myeloperoxidase; PBS = phosphate-buffered saline; pSS = primary Sjögren's syndrome; RA = rheumatoid arthritis; RT-PCR = reverse transcriptase polymerase chain reaction; SGECs = salivary gland epithelial cells; SLE = systemic lupus erythematosus; TNF = tumor necrosis factor.

## Competing interests

The authors declare that they have no competing interests.

## Authors' contributions

MI performed cultures of salivary gland epithelial cells, conducted the treatment, detection and quantification of BAFF secretion, real-time quantitative RT-PCR, and participated in the study design and drafting of the manuscript. CM performed real-time quantitative RT-PCR and participated in the study design and drafting of the manuscript. JEG performed statistical analysis and participated in the study design and drafting of the manuscript. FL and JS participated in the study design and drafting of the manuscript. TL and NB performed immunocytochemistry experiments. CL performed the biopsies of minor salivary glands. XM conceived of the study and its design and edited the manuscript. All authors read and approved the final version.

## Figures and Tables

**Table 1:** Modulation of BAFF mRNA expression with stimulation by cytokines in seven patients with pSS and seven controls

Condition	Patients with pSS (*n *= 7)			Controls (*n *= 7)		
	BAFF mRNA/β-actin mRNA	Fold increase over baseline	*p *versus baseline^a^	BAFF mRNA/β-actin mRNA	Fold increase over baseline	*p *versus baseline^a^
Baseline	0.44 ± 0.08			0.58 ± 0.2		
IL-10	0.9 ± 0.36	3.38	0.3	0.7 ± 0.28	1.57	0.3
TNF-α	0.7 ± 0.28	1.62	0.27	1.0 ± 0.3	3.15	0.25
IFN-α	9.2 ± 2.47	26.96	**0.03**	13.7 ± 9.1	19.06	0.2
IFN-γ	10.4 ± 1.65	25.01	**0.008**	12.2 ± 4.37	26.65	**0.03**
IFN-γ + TNF-α	23.7 ± 6.86	62.06	**0.008**	8.3 ± 3.18	17.66	0.06

^a^Paired *t *test; bold values indicate significant difference (*p *< 0.05).

Ratios of BAFF mRNA to β-actin mRNA are expressed as means ± SEM. BAFF = B cell-activating factor; TNF = tumor necrosis factor.
